# In-orbit demonstration of a re-trainable machine learning payload for processing optical imagery

**DOI:** 10.1038/s41598-023-34436-w

**Published:** 2023-06-27

**Authors:** Gonzalo Mateo-Garcia, Josh Veitch-Michaelis, Cormac Purcell, Nicolas Longepe, Simon Reid, Alice Anlind, Fredrik Bruhn, James Parr, Pierre Philippe Mathieu

**Affiliations:** 1 Trillium Technologies Ltd., 27-29 South Lambeth Road, London, SW8 1SZ UK; 2 Image Processing Laboratory, University of Valencia, Valencia, Spain; 3 Department of Computer Science, ETH Zurich, Zurich, Switzerland; 4 School of Computer Science and Engineering, University of New South Wales (UNSW), Sydney, Australia; 5 Phi-Lab Explore Office, European Space Agency (ESA), Frascati, Italy; 6 D-Orbit SpA, Viale Risorgimento, 57, 22073 Fino Mornasco, Como Italy; 7 Unibap AB (Publ.), Kungsängsgatan 12, 753 22 Uppsala, Sweden

**Keywords:** Hydrology, Natural hazards, Engineering

## Abstract

Cognitive cloud computing in space (3CS) describes a new frontier of space innovation powered by Artificial Intelligence, enabling an explosion of new applications in observing our planet and enabling deep space exploration. In this framework, machine learning (ML) payloads—isolated software capable of extracting high level information from onboard sensors—are key to accomplish this vision. In this work we demonstrate, in a satellite deployed in orbit, a ML payload called ‘WorldFloods’ that is able to send compressed flood maps from sensed images. In particular, we perform a set of experiments to: (1) compare different segmentation models on different processing variables critical for onboard deployment, (2) show that we can produce, onboard, vectorised polygons delineating the detected flood water from a full Sentinel-2 tile, (3) retrain the model with few images of the onboard sensor downlinked to Earth and (4) demonstrate that this new model can be uplinked to the satellite and run on new images acquired by its camera. Overall our work demonstrates that ML-based models deployed in orbit can be updated if new information is available, paving the way for agile integration of onboard and onground processing and “on the fly” continuous learning.

## Introduction

In recent years, machine learning (ML) and deep neural networks in particular, have boosted the possibilities of ground-based analysis of Earth-observation data. Many recent works have shown that much of the previously labour-intensive remote-sensing work can be automated in a robust fashion with ML. Examples include infrastructure delineation (such as buildings^1,2^, ships^3^, schools^4^ or solar panels^5^), agricultural applications^6^ or disaster response^7,8^. Machine learning techniques are able to exploit large stacks of data to derive meaningful products and realistic uncertainties. However, these capabilities come with very large overheads in ground-based computing power, training time and data-transfer costs. It is also increasingly recognised that machine learning models are difficult to generalise—that is, to apply outside of the context or domain in which they were trained, such as to a different areas of the Earth, or to images acquired under different conditions (for example with a slightly different sensor or after atmospheric properties have changed)^9^. Additionally, the performance of ML models degrades over time as the real world changes. This means that new data must frequently be added to the mix and models re-trained at a high cadence, which can be a costly exercise.

A revolution in satellite technology is happening in parallel. Constellations of small satellites are constantly adding to the total volume of Earth-observation data being collected each day. More satellites are being launched every month, leading to an explosion in data, much of which is complementary to the imagery gathered by the European Space Agency (ESA) Copernicus program. Small, independent satellites could serve different purposes such as filling gaps of between revisits, capturing images in alternative wavebands to increase spectral coverage, drawing attention to events that should be imaged in detail, or rapidly sending critical information to the ground. If properly organised, this ensemble population of orbiting devices could also act in concert: sharing data, processing power and sensors in orbit, leading to an overall greater capability. This benefit is not currently realised because of a lack of cooperation between satellites and a deficit of onboard intelligence, which is critical to perform advanced analysis and to coordinate group actions. Downloading data from orbit is also challenging and costly because of limited communication bandwidth and the difficulty of coordinating available ground stations.

We believe that the solution to the linked challenges of high download and re-training costs, imperfect observational coverage, post-analysis model drift and coordination issues is to build significant perceptual capabilities into loose networks of satellites using ML. Such onboard intelligence could help automate analysis in orbit so that only high-level (and likely smaller) data products are downloaded. The recent development of *federated learning*^10^ seems tailor-made for such operations, as it is designed to process data at the ‘edge’ of the network, with intelligent devices only sharing representations of what they have learned. In this manner, data-fusion could be achieved by directly sharing representations between trusted instruments so that satellites, large and small, can cooperate as a true hybrid system.

The concept of a ‘ML payload’ is a central idea necessary to accomplish this vision. A ML payload is a self-contained machine learning software module that produces advanced products—insights or learned representations—from raw observed data. Very recently Giuffrida et al.^11^ demonstrated a ML payload implementing a cloud detection deep learning based algorithm that was tested onboard the $\Phi$-Sat-1 satellite. They showed how this module could be used to discard overly cloudy scenes, saving expensive communication bandwidth.

In this work we demonstrate—onboard a satellite deployed to low Earth orbit (LEO)—a ML payload capable of sending compressed data products (vectorised flood maps) based on the *WorldFloods* work^12^. We compare the performance of different model architectures at effectively mapping water in Earth observation (EO) data and demonstrate that we can produce vectorised outputs from full Sentinel-2 acquisitions, detecting water within the constrains of the mission (satellite hardware, processing time and size of derived outputs). We also show that the ML payload can operate on images from the onboard ‘D-Sense’ RGB camera. For the first time, we demonstrate a successful update of the ML model to adapt it to this camera after the satellite was launched. In particular, we re-trained the model on the ground using images acquired by the camera and uploaded the new compressed weights back to the satellite to be used by the payload. This capability is crucial for ML payloads to adapt to new sensing instruments and continually address data-shift problems.

The rest of the paper is organized as follows: in “Methodology” section we describe the engineering of this system and the constrains of the mission, in “Experimental setup” section we state the goals of the mission and describe the experimental setup. “Results and discussion” section presents results from a comprehensive benchmark test of the models in different representative edge devices, while the results of the payload execution in orbit are presented in “Conclusions” section.

## Methodology

### The ML payload

The first and simplest component of a machine learning enabled satellite network is the ML payload. This is a self-contained machine learning software module that can be considered analogous to a hardware payload, such as a camera or sensor. ML payloads are typically encapsulated in a virtualised software container, isolated from the base computing environment and adjacent software. *PodMan*^13^ and *Docker*^14^ are two of the most popular virtualisation frameworks that enable guest operating systems with custom environments to be distributed as single ‘image’ files, with all dependencies included. Usually containers are compiled in a layered fashion, such that multiple containers can extend a shared ‘base’ image that contains common dependencies. This greatly simplifies the process of developing software for onboard processing, allowing the satellite computing module to offer a familiar and consistent base system (e.g., a standard linux-based tool-chain and Python software stack), with hardware and data access exposed through a simple application programming interface (API).

ML payloads also offer a simple pathway to upgrade, correct or enhance satellite capabilities in a relatively risk-free way. For example, neural networks can be re-trained to perform better (e.g., by utilizing newly available data, taking advantage of new acquisition parameterisations, or adapting to the specifics of a new sensor, or in response to previously unseen events), or even to recognise more classes of terrain in images. At a minimum, only the weights of the network need to be altered and the pre-validated supporting software stack can be left unchanged. Hence, the risk of introducing bugs due to code-changes is ameliorated. Network weight definitions are also significantly smaller in size ($\sim$ 1–20 MB) than a full software stack, meaning much lower upload costs.

**Figure 1:**
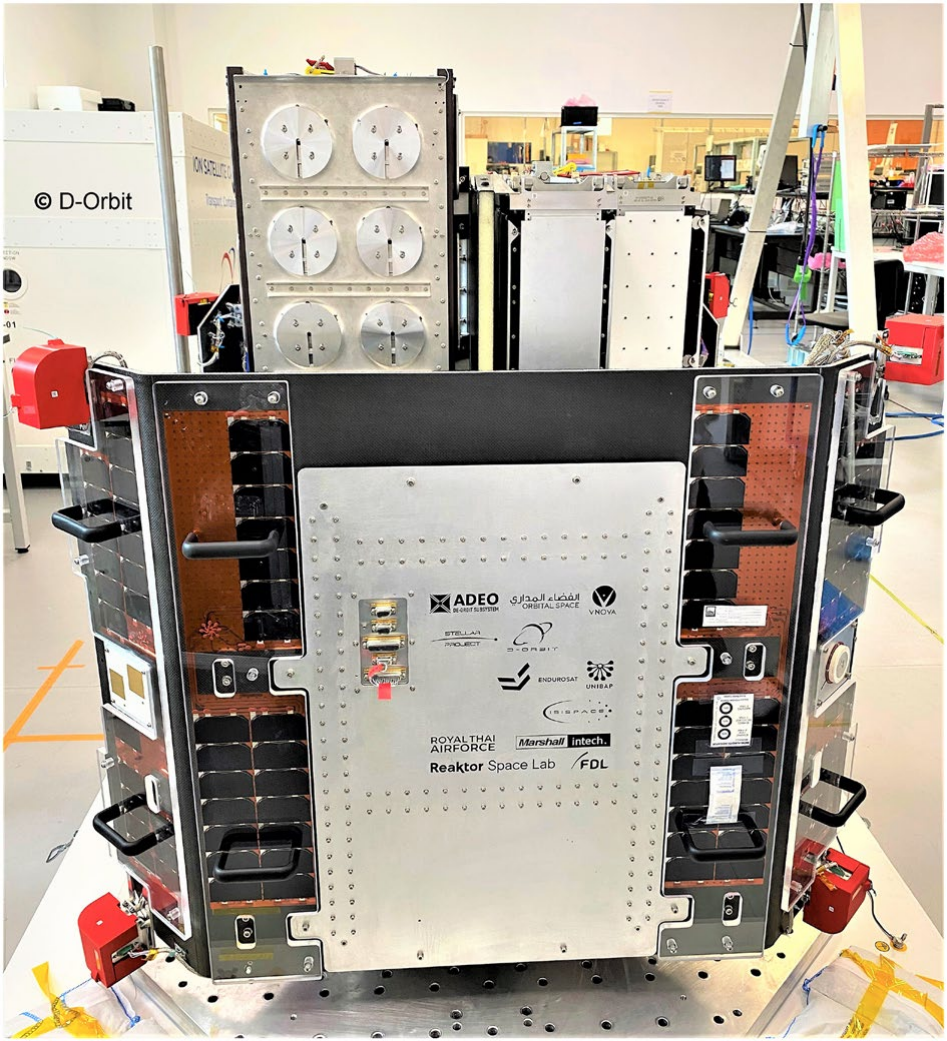
Picture of the D-Orbit ION Satellite Carrier *Dauntless David* being prepared for launch. After reaching LEO on board a SpaceX Falcon 9 rocket the satellite disconnects and ferries client SmallSats into custom orbits. The satellite also carries internal hardware payloads, one of which is D-Orbit’s Cloud Computing in Space module, which is used to run the *WorldFloods* ML payload. Image used with permission of D-Orbit.

### The Wild Ride mission: a ML payload testbed

Trillium has partnered with D-Orbit^15^, Unibap^16^ and ESA $\Phi$-Lab^17^ to build and test a ML payload on a prototype satellite constellation node. D-Orbit is a space logistics and transportation company offering MicroSat and CubeSat deployment services through their ION Satellite Carrier^18^. The D-Orbit *Wild Ride* mission for the carrier *ION SCV Dauntless David* successfully launched into LEO on a SpaceX Falcon 9 rocket on June 30th 2021 (see Fig. 1). In addition to seven satellites destined for deployment to multiple orbits, the carrier also included three internal demonstrator payloads, including D-Orbit’s Cloud Computing in Space module—the first iteration of an on-orbit cloud computing module being developed by Unibap.

The Cloud Computing in Space module can be considered a precursor to a fully-fledged space cloud node, offering a quad-core x86 64-bit processor, a Microsemi SmartFusion2 FPGA and an Intel Movidius Myriad X Vision Processing Unit (VPU). In particular, the onboard Myriad X processor accelerates machine learning inference and makes it possible to deploy deep artificial neural networks (ANNs) in a power-constrained environment (1 TFLOPs of compute with a nominal consumption of 1W^11^). The Myriad X chip underwent a radiation characterisation in ESA test facilities and has already been tested in space on the $\Phi$-Sat-1 mission^11^. The module also carries the D-Sense sensor module^19^, which includes a basic RGB camera, similar to a standard webcam. *Dauntless David* will remain in low Earth orbit for approximately two years, conducting engineering tests and experiments.

**Figure 2:**
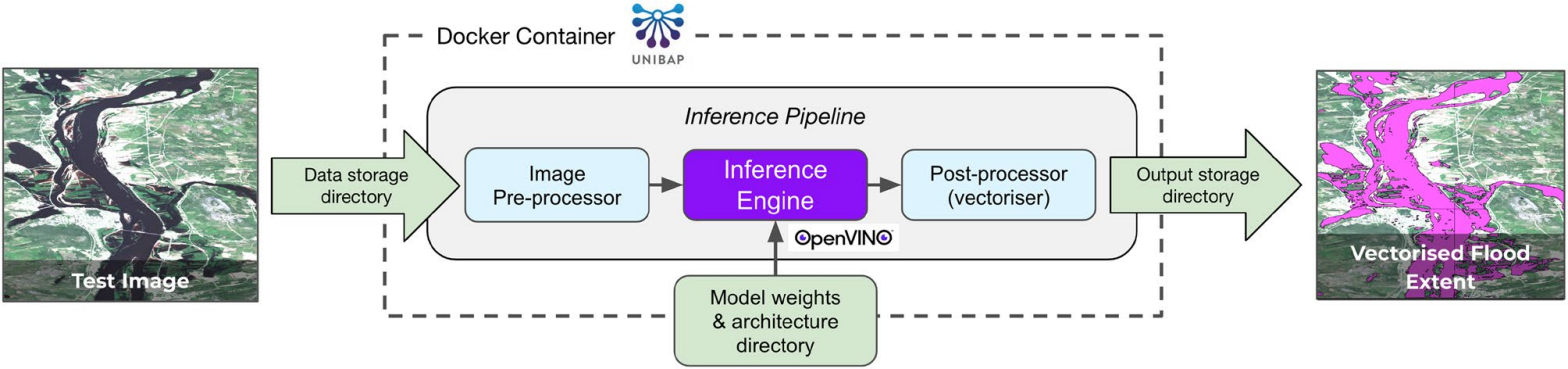
Schematic diagram of the *WorldFloods* ML payload for the Unibap SpaceCloud Framework. The inference pipeline is built in Python and uses the Intel OpenVINO Inference Engine for the Myriad X processor. The application is contained within a *Docker* environment and accesses data in externally mounted directories, colour-coded green here. The SpaceCloud Framework manages sensor access and communications, and provides a standard Linux/GNU computing environment, which greatly simplifies the development process.

### The WorldFloods ML payload

For this project we chose to deploy the *WorldFloods* ML payload, which was developed in partnership with ESA during Frontier Development Lab (FDL) Europe 2019^20^. *WorldFloods*^12^ is a comprehensive dataset and suite of machine learning models that can be used to create flood masks from multi-spectral Earth-observation images. The segmentation models can distinguish between cloud, land and water, and were trained on multi-band images from ESA’s Sentinel-2 (S2) satellite, including the infrared bands.

The multi-spectral instrument of S2 is a push-broom sensor with high radiometric resolution (12-bit). Its spectral response covers the visible, near-infrared and shortwave-infrared ranges (490–2380 nm) with 13 bands, with a spatial resolution varying from 10 to 60 m depending on the band. In this work we re-sampled all the bands to 10 m which is the resolution of the visible and infrared bands. As in Mateo-Garcia et al.^12^, we used level 1C S2 products. Level 1C products are processed to calibrated top-of-atmosphere reflectances and the images are geo-referenced, and ortho-rectified (see the S2 User Handbook for details^21^).

When deployed on a satellite, *WorldFloods* offers an enhanced ability to rapidly map the spatial extent of water bodies and flooding detected by orbital sensors. At present, creating a flood map at sufficient resolution for first responders ($\sim$10 m) can take up to 48 hours due to the lead time involved in downloading, processing and interpreting high-resolution multi-spectral data, followed by transmitting the derived maps to the disaster zone. If the multi-spectral data can be processed in orbit instead, a vectorised polygonal outline of the flooded region could quickly be transmitted to the ground. This data product is potentially tens of times smaller in size, making it feasible to push directly onto mobile devices in the field—within minutes of being acquired. At present, the cost of downloading data from orbit dominates most operational budgets, so even modest decreases in file size offer potentially significant savings.

**Table 1:** Different models tested to segment flood water in Sentinel-2 images.

Model Name	Description	# Params	Model Size	
			PyTorch	IR
NDWI	Normalised Difference Water Index—a baseline method of enhancing the contrast of water by comparing relative colour of the green (B3) and the infrared band (B8) of Sentinel-2 ^22^.	NA	NA	NA
MNDWI	Modified Normalised Difference Water Index—similar method using a short-wave infrarred band (B11) of Sentinel-2 instead of B8 which is known to be more accurate for flooding water detection ^23^.	NA	NA	NA
Linear	A simple 1-layer fully-connected neural network.	42	4 KB	20 KB
SCNN	Simple CNN. A lightweight convolutional neural network (CNN) with only five convolutional layers. Architecture proposed in^12^.	0.26M	1 MB	540 KB
UNet	A fully convolutional neural network first proposed in Ronneberger et al. ^24^ stacking up-sampling layers after the standard down-sampling convolutional blocks.	7.8M	30 MB	15 MB
HRNet	A recent multi-branch architecture proposed in Sun et al. ^25^ that retains high-resolution representations of the input data all the way through the network.	3.8M	16 MB	8.3 MB

Here, IR stands for Intermediate Representation, a compiled model format used for edge deployment. Most of these models were proposed in Mateo-Garcia et al. ^12^. Models ordered in increasing order of complexity. The PyTorch implementation of all the models is open-sourced at https://github.com/spaceml-org/ml4floods.

### Model development

The *WorldFloods* segmentation models created during FDL Europe 2019^12^ have recently been open-sourced in a public python package called ‘ML4Floods’^26^. In this framework, users can train segmentation models using the *WorldFloods* dataset and different S2 band combinations. These models can subsequently be benchmarked using a dedicated set of test images from *WorldFloods*, or applied to new S2 images that can also be downloaded with the assistance of the ML4Floods package. For this work, we use the models with all thirteen S2 bands published in Mateo-Garcia et al.^12^, but we also train new model versions using only three visible bands B2 - B4, to approximate a standard RGB camera (e.g., like the D-Sense camera on the compute module). It is well-known that infrared (IR) and short-wave infrared (SWIR) bands are the dominant discriminators of water in optical EO data^23,27,28^, so we expect the RGB-only models to perform worse than multi-band models. In “Results and discussion” section we directly compare the performance of the RGB models against the all-band models.

The available model variants are presented in Table 1. The architectures of the Linear, Simple CNN and U-Net models are the same as presented in Mateo-Garcia et al.^12^, but we added HRNet^25,29,30^ as an example of a modern architecture that has produced state-of-the-art results in several semantic segmentation tasks, including remote sensing problems (see e.g., Etten and Horgan^1^). The implementation of all the model-training pipelines is open-sourced in the ML4Floods GitHub package^31^.

### Adapting models to the D-Sense camera after the satellite launch

As we previously highlighted, it is well-known that ML models struggle when they are applied outside the context in which they were trained. In the ML literature this problem is known as *domain-shift*, or *data-shift*^9,32^, and it occurs when the distribution of the data is different at training and testing times. In the context of remote sensing, this is a conspicuous problem that arises every time a model (ML-based or otherwise) developed for one sensor is applied to another with slightly different characteristics (radiometric shift), or to a previously unseen area (geographical shift), or to images observed through different atmospheric conditions (seasonal shift). In our case, we observe this problem when the *WorldFloods* models (trained on calibrated S2 images, 10 m resolution, 12-bit depth) are applied to images taken by the D-Sense camera ($\sim$1 km resolution, 8-bit depth, no calibration and significantly worse radiometric quality). We show in “Results and discussion” section that indeed the differences between the images lead to very poor model transfer performance.

There are inter-calibration and domain adaptation techniques^33^ that could potentially address this problem and do not require supervised information for the D-Sense sensor. These techniques attempt to align the colour and size distributions of the two domains (S2 and D-Sense) so that a model trained with supervised information from S2 images could work on D-Sense images. We initially tried histogram matching^34^, which seeks to align the color distributions of the two sensors—but without success. We also attempted to retrain the models on down-scaled S2 images made to resemble the spatial resolution of the D-Sense camera (as proposed in^12,35^). However, the segmentation results were still unsatisfactory and therefore we did not try more advanced domain adaptation methods (e.g., Mateo-Garcia et al.^36^ or Tasar et al.^37^).

Hence, in order to build a sufficiently good model for processing D-Sense camera data, we incorporated supervised information on native D-Sense images. To build a training dataset we downloaded four D-Sense acquisitions of the Earth (size $2500\times 1950$ pixels) and annotated regions of water, land and cloud with manually drawn polygons. We trained new models to segment D-Sense images, both by using the S2 RGB *WorldFloods* models as a starting point (called ‘fine-tuning’ in the literature) and by training from randomly initialised weights. The performance of the SCNN model displayed the best trade-off in accuracy vs model size and was chosen for uplinking to the satellite. We present the validation metrics of all models and some representative examples in “Results and discussion” section.

### Engineering the ML payload

The ML4Floods Python toolbox produces trained network definitions and weights in the PyTorch format, and these comprised our starting point. The PyTorch files must be converted to the Intel OpenVINO intermediate representation (IR) format to run in the Myriad X chip. This conversion process quantises the weights and intermediate tensor representations to 16 bit floats (FP16 or ‘half precision’), shrinking the size of the weights on-disk file size and speeding up inference in the Myriad X processor. Table 1 shows the size of the model definition files, which vary between 8 KB and 15 MB for the quantized versions in IR format. Deploying these models on the Unibap SpaceCloud hardware required further development steps:

- Finalise and test the tool chain to convert models from PyTorch to IR format via the Open Neural Network eXchange (ONNX) format.
- Build an inference pipeline that ingests a multi-band image and produces a vectorised mask outlining cloud, land and water.
- Encapsulate the inference pipeline in a ML payload software container and integrate into the Unibap SpaceCloud Framework.
- Test and tune the ML Payload so that it functions within the processing envelope of the hardware for the mission: a wall-time under 600 s and using less than 2 GB of memory.

The Unibap SpaceCloud Framework (SCFW) is a software platform running on the satellite payload computer and providing a *Docker* host for deploying custom containerised applications. The SCFW abstracts access to satellite sensors and application management routines via a simple API that supports multiple languages via protocol buffer definitions. The containerised environment is based on Ubuntu Linux (for this mission, version 18.04), meaning that SCFW applications can be developed on commodity x86 hardware using popular languages, rather than specialised languages designed for embedded programming. This system greatly accelerated development, which took place over $\sim 4$ weeks during May–June 2021.

Our *WorldFloods* payload application targeted the Myriad X processor to speed up machine learning, meaning that it was restricted to using the inference engine provided by the Intel OpenVINO Toolkit^38^. However, this proved to be a boon as the inference engine can be called from the Python language in which previous development had been done. Myriad X processors are also readily available off-the-shelf with USB interfaces^39^ so testing of network architectures could be done directly on the target hardware—essential for space-qualifying the ML payload.

A schematic diagram of the ML payload is shown in Fig. 2. As a prototype SCFW application, it is currently designed to be triggered from the ground when data becomes available in the input directory. The application detects and normalises the data cubes (depending on the pre-processing required by the requested model) and then pushes the data through the neural network in a forward pass, producing spatial per-pixel masks that classify the image into ‘land’, ‘cloud’ and ‘water’ categories. These intermediate pixel masks are written to a temporary directory before being further processed into polygonal mask outlines. The integer masks are converted to polygons using the *rasterio* python module, which offers a suitable algorithm in the ‘features.shapes()’ method. Under the hood, the method calls the C routine ‘GDALPolygonize‘ of the Geospatial Data Abstraction Library (GDAL)^40^. This vectorisation process effectively compresses the mask information, with the loss of some fidelity, although the balance between resolution and compression can be tuned. We initially saved the polygons to disk as plain-text files of vertices, but later found that the binary GeoPackage format produces a significantly smaller file. These GeoPackage (.gpkg) files are compressed together with some logging information and written to an output directory, which is queued for syncing with ground-based servers.

Mission parameters impose a memory limit of 2 GB and a maximum contiguous processing time of 600 s. The first version of our application significantly surpassed both of these limits when processing the large $10\times 10$ k pixel S2 chips. To solve the memory problem we sliced each data cube into multiple overlapping ‘tiles’ of $256 \times 256$ pixels, performed inference on each of these separately and sequentially updated a full-chip pixel mask on storage. To tile and stitch the predictions we followed the recommendations of Huang et al.^41^ by making predictions with overlap (16 pixels) and discarding the predictions at the borders of the tiles (this prioritizes predictions with larger receptive fields). To overcome the memory limitations, we used memory mapping to iteratively build the full pixel mask on disk. For the vectorisation step we similarly divided this full-chip pixel mask into overlapping tiles (this time with a larger tile size of 1,$024 \times 1$,024 pixels). To work around the processing time limit, we instructed the application to stop and save its state when approaching the cutoff time. On the next processing window, the application would pick-up where it left off to complete the analysis, setting a ‘done’ flag in the output directory when complete. The final masks and meta-data were then compressed into a ZIP file, ready for download.

The ML payload application is controlled by running a custom *Docker* command and feeding the controlling script with different parameters. These specify the file system directories to access input and output data, the model name and weight definition directory, the processor device (e.g., Myriad X or CPU) and the processing time-limit. Different experiments can be performed by changing these inputs and—crucially—the application can be pointed towards completely new weight definition files, allowing the models to be updated without significant infrastructure changes.

## Experimental setup

The original goal of this work was to assess the practical use of machine learning on a small satellite to act as an ‘outrigger’ processor for ESA Copernicus Sentinel-2. We also want to prove that an orbiting ML payload can be updated with new weights to improve performance and to adapt to a custom instrument. Specifically, the goals of the project are:

1. Benchmark the *WorldFloods* ML payload on operational hardware with a Myriad X processor and on other similar edge devices.
2. Demonstrate that the ML payload can successfully create water masks of a full S2 chip ($10\times$10 k pixels, 13 spectral bands) within the mission constraints.
3. Re-train the *WorldFloods* models to perform inference on the lower resolution RGB-only data supplied by the D-Orbit D-Sense camera.
4. Demonstrate that the new network weights can be successfully uploaded to the satellite and that the upgraded ML payload can be deployed to create water masks from the RGB D-Sense images.

A progressive validation process for the ML Payload was implemented to test the developed software. Firstly, the payload was tested locally on our development machines using USB-format Myriad X devices. Once our tests indicated that the ML payload was running with the imposed constrains we were given access to a SpaceCloud test device (a ‘flatsat’) with similar hardware characteristics as the computing payload. Using these machines, we ran a comprehensive suite of benchmarks to obtain performance statistics of the different models. Finally, based on those benchmarks, we selected the models and the experiments to run in orbit.

For benchmarking the models, we compare four critical variables: *processing time* (which includes the inference time of the network plus the time taken to vectorise the resultant water mask), the *compression ratio* (ratio between the size on disk of the input image and the vectorised output product), the *Intersection over Union* (IoU) of the water mask—this is the metric to measure the accuracy of segmentations used in^12^—and the *weight size* in bytes, which is a critical cost factor when uploading the model back to the satellite.

**Figure 3:**
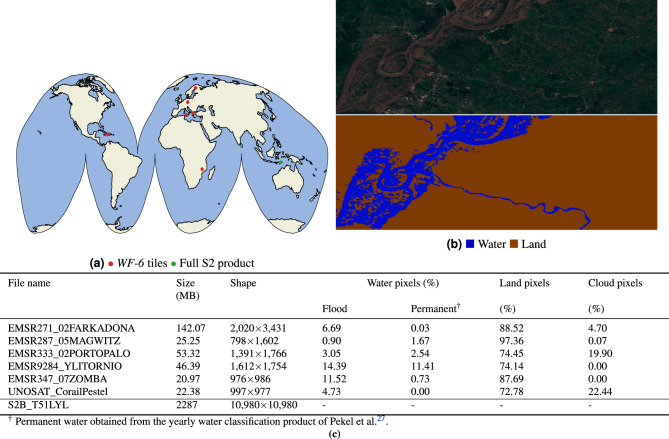
(**a**) Location of the S2 tiles used for testing the payload. In red the six products from the *WorldFloods* dataset (called *WF-6* dataset); in green the full S2 acquisition. (**b**) RGB sample of one of the *WF-6* products with its ground truth mask. (**c**) Statistics of size, shape and class of the pixels of the *WF-6* products and the full S2 acquisition.

### Experimental data

For Sentinel-2, models were trained on the training split of the *WorldFloods* dataset (see Mateo-García et al 2021^12^). To test and benchmark our ML Payload on Sentinel-2 imagery we selected six flood products from the *WorldFloods* validation and testing dataset consisting of an image and its corresponding ground truth mask (see Fig. 3). We refer to these products as the *WF-6* dataset and these were chosen to be of similar size and to sample a range of different surface conditions. As presented in Mateo-García et al.^12^, training and test images are from different flooding events and there is no spatial overlap between them. Additionally, we also selected a full S2 product (10,980 $\times$ 10,980 pixels) to measure the current processing capabilities of the payload for its operational use-case. Figure 3 shows the location of these and an example of a product with its ground truth mask. All these products were pre-loaded in the computing payload to test the system in-orbit.

**Figure 4:**
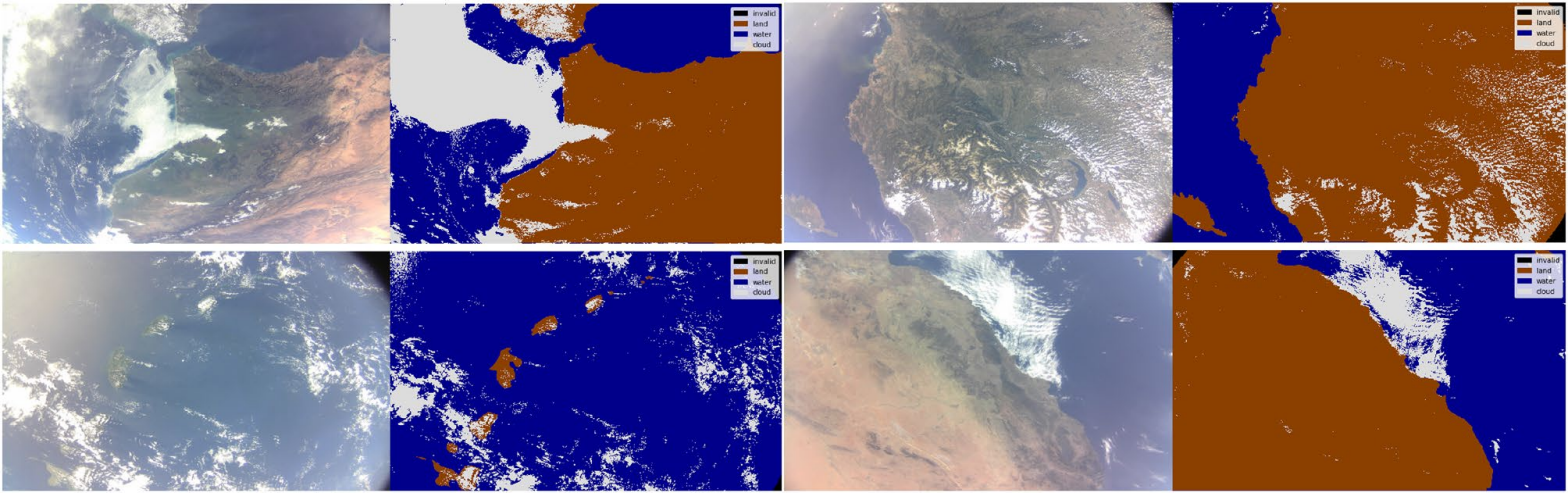
Images acquired by the D-Sense camera onboard the satellite, with their manually annotated label masks. These images are used for re-training the models using a leave-one-out validation scheme. We refer to this dataset as *D-Sense-4*.

Four D-Sense camera acquisitions were downlinked by the D-Orbit and UNIBAP teams to support re-training of models for that sensor. We manually labelled these scenes with *cloud*, *water* and *land* classes (i.e., pixel masks) using open-source computer vision software^42^. Figure 4 shows the acquisitions together with the manually derived masks, hereafter referred to as the *D-Sense-4* dataset. During training we follow the same approach as in Mateo-Garcia et al.^12^: we divide these large acquisitions into at total of 5048 overlapping tiles of 256 $\times$ 256 pixels each, which are sampled in mini-batches to feed to the training loop. In order to test the generalisation capabilities of those models we used a *leave-one-acquisition-out* scheme (LOO); that is, we iteratively train with three out of the four D-Sense image and test the model on the remaining one.

## Results and discussion

In this section we present the experimental results of the *WorldFloods* ML Payload and discuss their implications. In “Results in Sentinel-2 flooding tiles” section, we dive into the results of processing the *WF-6* dataset and compare different critical variables affecting operations on Unibap SpaceCloud hardware. In “Results in full Sentinel-2 acquistions” section we present the results of processing a full S2 product, both on the ground-based ‘flatsat’ and in-orbit, demonstrating that the inference time is within reasonable constraints. In “Results on D-Sense images” section we show the results of re-training of the ML Payload for the D-Sense camera, comparing the models trained on *WorldFloods* with those trained on the labeled *D-Sense-4* dataset. Additionally, we show the segmentation results of one of these models run on D-Orbit’s Cloud Computing in Space module, in orbit. Finally, we conclude this section with some complementary benchmarking experiments, showing the performance of the payload on other edge devices (“Model quantisation for edge devices” section).

### Results in Sentinel-2 flooding tiles

**Figure 5:**
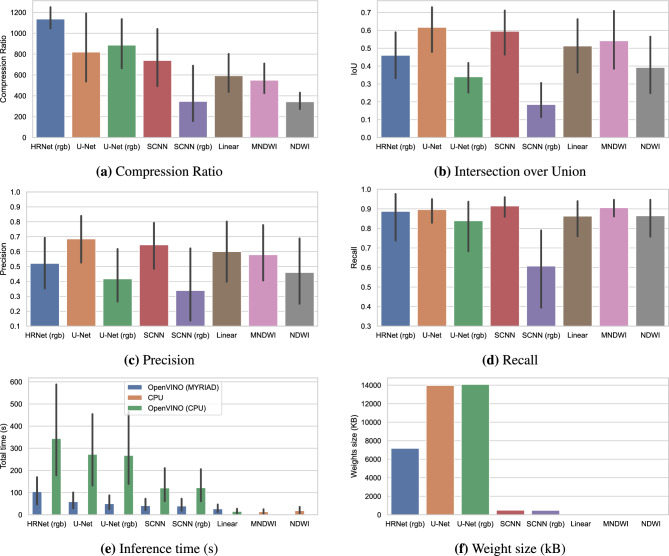
Averaged statistics of the different models over the *WF-6* dataset. This benchmark was run on a SpaceCloud device with an Intel X5 processor and a Intel Movidius Myriad2 chip. (**a**) Compression ratio of the final vectorised products (input size divided by output size) produced by the different ML models and baselines. (**b–d**) Intersection over Union, precision and recall of those models measured against the ground truth of the *WorldFloods* dataset^12^. (**e**) Total time (inference and vectorization) of the models running on different hardware. (**f**) Size of the weights in KB; this size is critical for updating the model after the satellite deployment.

Figure 5 illustrates the performance of our models (Table 1) applied to the *WF-6* dataset. In the top-left plot (a) we see that the compression ratio is between 400 and 800 in these products and tends to be greater for the more complex CNN architectures than for the baseline models (Linear, NDWI and MNDWI). This indicates that the polygons created by convolutional models are less complex (i.e., have fewer vertices) than the baselines. We hypothesize that this is because CNN models are able to understand the spatial context to produce simplified *as-human-drawn* polygons whereas the baselines, working in a pixelwise mode, produce more ‘salt-and-pepper’ (i.e., noisy) outputs, resulting in more complex polygons. The top-right plot (b) in Fig. 5 shows the averaged intersection-over-union of the predicted versus ground-truth water mask for the *WF-6* images. We see that models using all S2 bands perform significantly better those that use only the visible bands (noted as ‘rgb’ in the figure). This is expected, since the infrared channels are a very good indicator of water due to its high absorbance in this part of the spectrum. This is also reflected in plots (c, d) which show the precision and recall of the models. Particularly, we see that models have high recall which indicate that they capture most of the flooding water of the scene. The plot in the bottom-left (e) shows the total processing time required to produce the products when each model is run in the SpaceCloud ‘flatsat’. We tested the CNN models running on the CPU and in the Myriad X chip via the OpenVINO inference engine, and the baseline (M)NDWI models running directly on the CPU. We see that the Myriad chip accelerates computing speed by factors of $3\times$ to $6\times$ and that total time (including inference and vectorisation) is lower than 1 min for all the models, except for the more complex HRNet architecture. This shows that without much optimization and specific hardware we can obtain vectorised results in less than 1 min for areas of around 1000 $\times$ 1000 pixels (see Fig. 3 for sizes of the *WF-6* products). Finally, the plot in the bottom-right (f) shows the on-disk size of the weight files in kilobytes. This is a critical variable for re-trainable payloads since the uplink capacity of the communication payload of the satellite is usually much lower than the downlink capacity. For the *Wild Ride* mission, the size of the uploaded packets must be under 500KB. This gives the SCNN model a huge advantage since its size is 16 to 28 times smaller than the HRNet and UNet, respectively.

**Figure 6:**
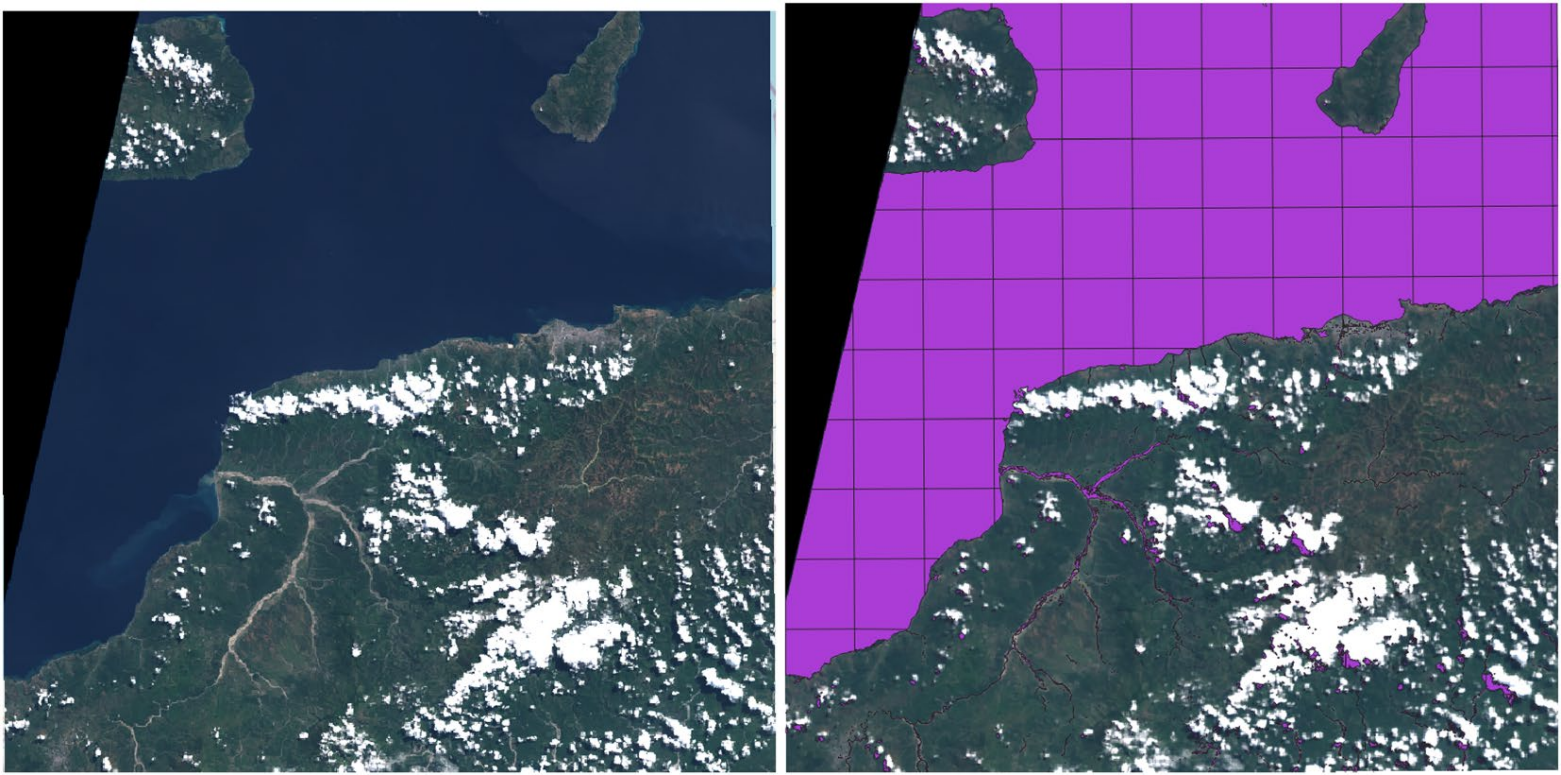
Left: Visible light RGB image of the full Copernicus Sentinel 2 chip that was pre-loaded onto the satellite for testing purposes. Sentinel 2 images are provided as public domain. Right: The water mask produced onboard by the linear model is overlaid as a purple-filled polygon that exhibits a particularly simple morphology, with fewer vertices than average. The grid-pattern on the water mask shows the processing tile boundaries. The image also includes a blank swath (in black) where there is no valid data. This chip was deliberately chosen to provide a high compression ratio, keeping data download costs to a minimum.

Before moving to the results in the full S2 acquisition, it is worth to take into account that the processing time may vary significantly between images of the same size. Inference times are consistent for similar sized tiles, but the time taken to vectorise the data depends heavily on the appearance and morphology of water and cloud in the field of view. A complex scene (e.g., Fig. 3b) will contain many more polygon vertices than a simple scene (e.g., Fig. 6) and the processing time scales with the number of vertices. Additionally, we found that the average compression ratio for these multi-band S2 chips is around $\sim 400$, but like processing time, the compression depends heavily on the complexity of the features present in the data.

### Results in full Sentinel-2 acquistions

Figure 6 shows the full $10,980\times 10,980$ pixel Copernicus S2 tile included on the computing payload (at left) and the same image overlaid with the vectorised segmentation mask produced on-board (at right). The ‘Linear’ model architecture was applied in this case. This image mask was iteratively processed in overlapping 256-pixel square tiles, sized to fit within the memory constraints imposed by the mission. The full predicted mask was subsequently vectorised—also by tiles, but in this case 1024 square pixels. These tiles can be seen in the square grid drawn over the water mask in the image at right. The resulting vector mask was compared with the output of the original PyTorch-based *WorldFloods* model and the agreement between masks is over 99.9% similar.

**Table 2:** Running time statistics of the different models over the selected full S2 product (10,980 $\times$ 10,980 pixels).

Model	Compression ratio	Inference time (s)	Vectorisation time (s)	Total time (s)
U-Net	9379.4	2421.7	17.3	2439.0
SCNN	9321.3	1643.7	17.4	1661.1
Linear	4874.0	1067.1	24.1	1091.2
MNDWI	2417.8	492.9	44.2	537.0
NDWI	1824.7	488.6	59.4	547.9
Linear (onboard)	4886.1	894.7	18.2	912.9

Models tested on UNIBAP SpaceCloud ‘flatsat’ except last row which was run onboard the satellite and whose results where downlinked to the ground.

Table 2 shows the processing time and compression ratio for this image segmented by different models on the Unibap SpaceCloud ‘flatsat’ and onboard the satellite (last row). For the SpaceCloud runs we see a similar picture as in previous figure, with U-Net and SCNN giving significantly higher compression ratios—near to 10,000. We can see that total processing time for the CNN models varies between 15 and 42 min, compared to approximately 8 min for the baseline (M)NDWI models. When we sub-divide the processing time into ‘inference time’ and ‘vectorisation time’ an interesting pattern emerges. As expected, inference time is much longer for the complicated CNN models ($\times 2-3$), even with the aid of the Myriad X accelerator. For these models, the total time is also dominated by the inference time (around 98% of the time of the SCNN model). However, the vectorisation step takes 2 - 3 times longer for the (M)NDWI models because of the more complex and ‘noisy’ masks and polygons they produce. Finally, when we compare the results of the Linear model run onboard and on the ‘flatsat’ twin, we see that the total time was significantly less (10% faster onboard) and that the compression ratio is slightly different. Nevertheless, when we compare the output products of the model run onboard and in the SpaceCloud ‘flatsat’ we see that the agreement of both masks is over 99.99%.

### Results on D-Sense images

**Figure 7:**
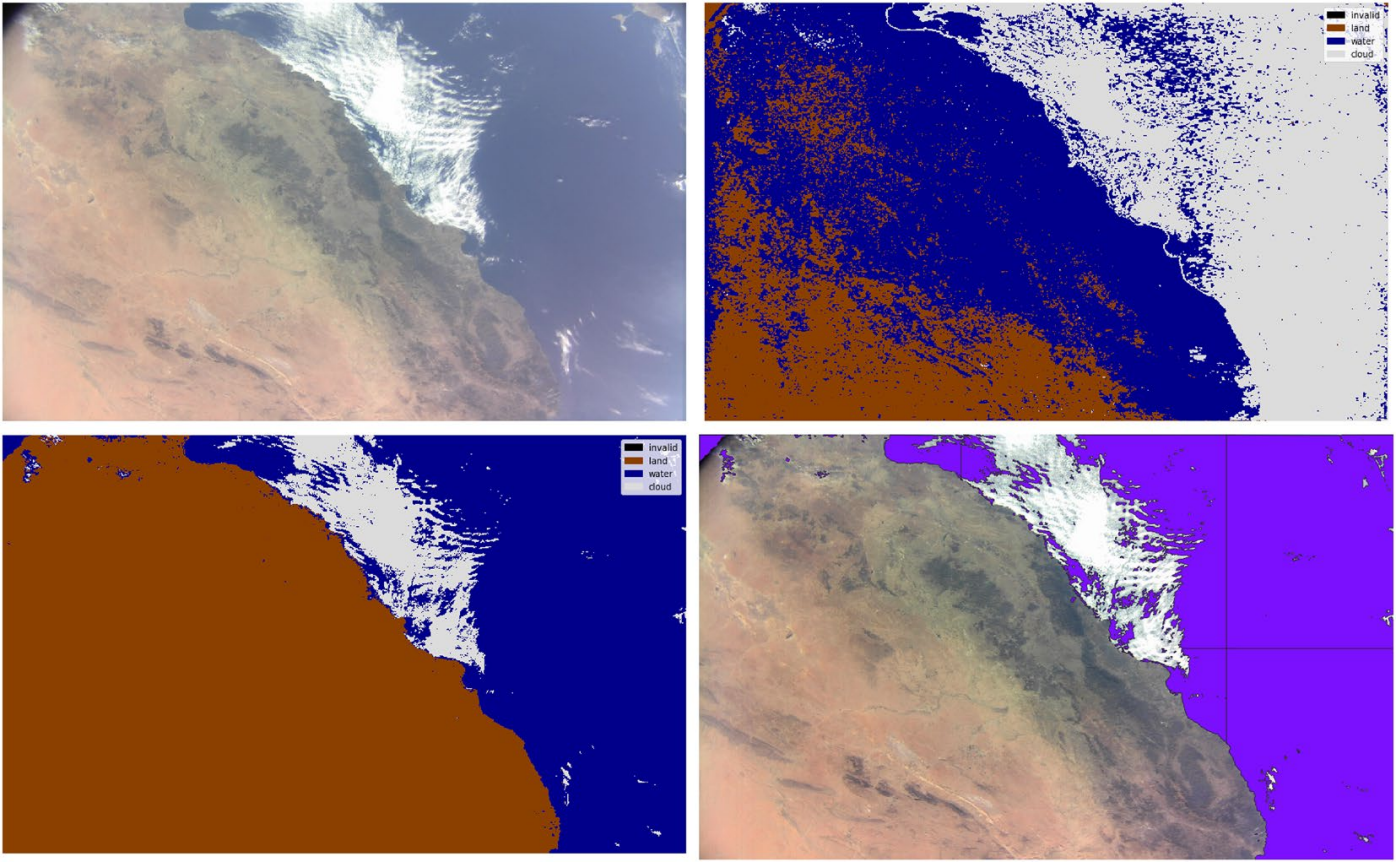
Top-left: Image from the RGB D-Sense camera. Top-right: Poorly performing mask produced by a SCNN model trained on *WorldFloods* using S2 data with RGB bands only. Bottom-left: High-performing mask produced by the SCNN model trained on D-Sense images. Bottom-right: Overlay of the vectorised water mask (in purple), from D-Sense trained model, on the image. This vector product has been generated onboard the satellite.

We showed in the previous section that the performance of the RGB models trained on the *WorldFloods* dataset is low (Fig. 5, top-right). This is likely because detecting flood water in RGB imagery is difficult due to the presence of debris and suspended matter that mainly affects the visible bands. This factor, together with the huge difference in resolution and radiometric quality between the S2 and D-Sense instruments, makes RGB models trained in *WorldFloods* perform poorly at detecting water in D-Sense images. Figure 7 shows a D-Sense acquisition (top left) and the results of applying the SCNN RGB model trained on the *WorldFloods* dataset. We see that this model produces mostly random noise as output.

It is worth mentioning that these results contrast with other deep learning models transferred across multispectral instruments that produced reasonable results. For instance, López-Puigdollers et al.^43^ and Mateo-Garcia et al.^44^ show that deep learning cloud-detection models trained on Landsat-8 work well when applied to Proba-V and S2 images. In our case, we hypothesize that the lack of calibration of the D-Sense camera together with the huge difference in spatial resolution and bad *per se* performance of the RGB models contributed to these results.

**Figure 8:**
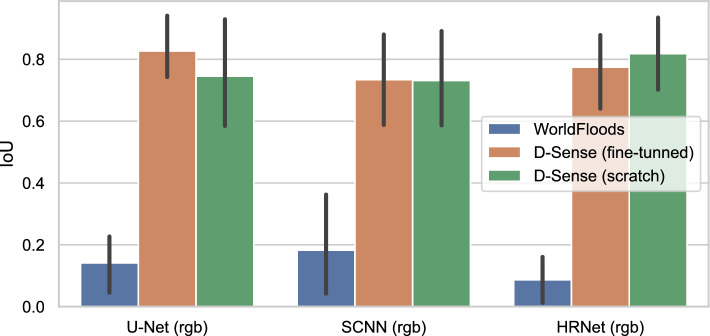
Intersection over Union (IoU) on the D-Sense labeled dataset (*D-Sense-4*) of the different models depending on the dataset used for training. The performance of the models trained in the *WorldFloods* dataset is very poor due to the differences between S2 and D-Sense images. When we train on the *D-Sense-4* data using a leave-one-out training scheme we obtain satisfactory segmentations. Training the models from scratch or fine-tuning the *WorldFloods* models produce similar results on D-Sense labeled data.

As outlined in “Experimental setup” section, we trained each model on the *D-Sense-4* data following a leave-one-image-out validation strategy. Figure 8 presents the intersection-over-union (IoU) for each RGB model, measured from the excluded images. We compare the models trained on *WorldFloods* (blue bars) against two different training strategies: fine-tuning from the pre-trained weights of the *WorldFloods* models and training from *scratch* (i.e., from randomly initialised weights). As we have seen before, the IoU of the original *WorldFloods* models is very low on D-Sense images. However, when those models are fine-tuned or trained from scratch on the small *D-Sense-4* dataset their IoU is comparable to, or even higher than, the *WorldFloods* models on S2 images reported previously (Fig. 5). Results in Fig. 8 show that models trained from scratch have similar segmentation accuracy to those trained by fine-tuning.

Finally, the two plots at the bottom of Fig. 7 show the output of the re-trained model (bottom left) and the vectorised water mask (in purple) overlayed on the unseen RGB D-Sense test image (bottom right). The segmentation result displays good accuracy overall, with some false-detections of water pixels at the top left of the image, where vignetting artefacts are evident. The vector mask shown in the bottom-left panel was obtained from the satellite after uploading the weights of the SCNN model to the ML payload. This result demonstrate, for the first time, that ML payloads can be updated *on-the-fly* after their deployment. We believe that this is a significant achievement that paves the way to future developments, since a mechanism to correct and continually improve the models after their operational deployment is crucial for ML onboard. Looking ahead, we envision future continuous learning systems that automatically deploy models onboard, that are trained on the ground, or in orbiting cloud computing services in a similar manner to Continuous integration/ continuous development (CI/CD) systems already existing in software development. CI/CD is a method to deliver software applications that introduces ongoing automation and continuous monitoring throughout the software lifecycle.

### Model quantisation for edge devices

Aside from the Myriad X, we have performed initial experiments converting our 13-channel models to run on several common edge-compute devices: the NVIDIA Jetson Nano (an ARM64-based single-board computer) and the Google Coral EdgeTPU.

The Jetson Nano is a low-cost computer with 128 onboard CUDA cores, sharing 4GB memory with the host CPU (ARM Cortex-A57). This allows for transferable testing of models trained in common machine learning libraries without much modification to code. We tested our models using PyTorch+CUDA as well as PyTorch bindings of TensorRT—NVIDIA’s hardware acceleration library designed for CUDA-capable devices. Generally, the limiting factor for running models is available RAM. Using the onboard CUDA cores, we were able to export a model capable of processing a $512\times 512$ pixel tile with the ‘Simple CNN’ model, which runs at around 1–2 tiles/s using standard PyTorch and 2–4 tiles/s when using the TensorRT back-end. While models with larger tile sizes may compile and run, inference is slowed down by the lack of RAM as the system begins to use swap memory. Our benchmarking also included testing on the ARM CPU, but inference latency was typically at least an order of magnitude slower than running on accelerator hardware.

Like the Myriad, the Google Coral EdgeTPU is an application-specific integrated circuit (ASIC) designed for neural network inference. In our testing device, we use a Mini-PCIe form factor card (model G650-04527-01) connected to the expansion port on the Jetson Nano developer kit. Model conversion requires two stages: we first export our model quantised to 8-bit precision; that is, the model uses 8-bit weights internally (versus floating point) and accepts 8-bit inputs. Next, we use Google’s EdgeTPU compiler to convert this model to a format that the accelerator can use. We were only able to export UNet models accepting up to a $256\times 256$ pixel images input due to RAM limitations. This UNet model has a processing time of around 10 tiles/s, but this is offset by the large number of tiles required (approximately 30k for a full chip) and processing takes around one hour. With a smaller architecture, like the Simple CNN we can compile up to $1024\times 1024$  pixel inputs. The inference time per tile is similar, but as there are far fewer tiles, processing a chip takes under 5 min, with vectorisation taking approximately 3–4 min for our test tile on the Jetson Nano.

**Figure 9:**
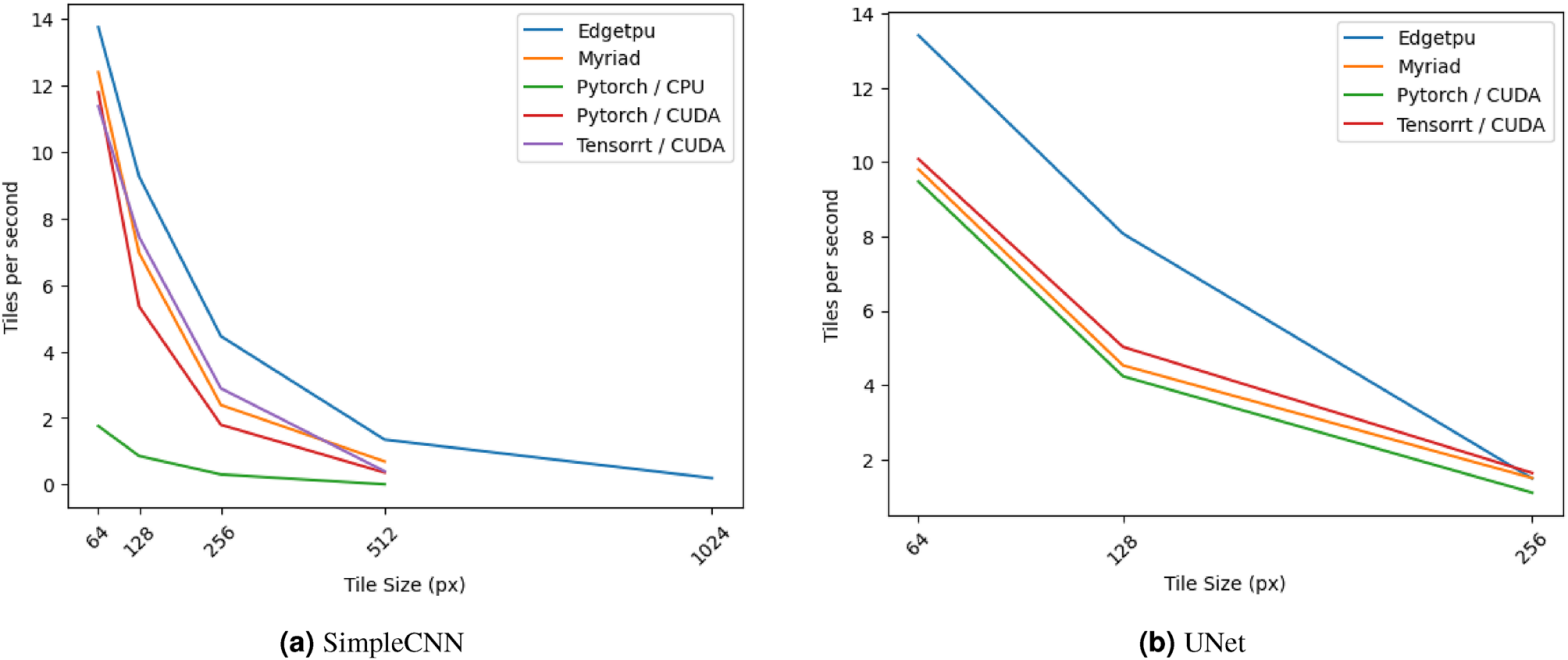
Inference latency results for (**a**) SimpleCNN and (**b**) UNet running on various edge devices connected to an NVIDIA Jetson Nano. We compare models accelerated in the Myriad-X chip, in the Coral EdgeTPU and using CUDA on the NVIDIA Jetson Nano (NVIDIA Maxwell with 128 CUDA cores). Results in Jetson Nano ARM CPU are ommited for UNet as they are an order of magnitude slower. Results are reported as tiles processed per second. While larger tiles may take longer to process, fewer tiles are required for a given image size. Where results for a particular framework and/or device are missing, a model was not available at that tile size due to memory limitations.

We were able to compile larger models for the Myriad, but we found that beyond tile sizes of 256 px, there was a significant delay during model load which exceeded the cutoff processing time available on the satellite. We therefore recommend benchmarking at a variety of tile sizes to select an optimal and practical value for the target hardware. In Fig. 9 we provide sample latency results on our test system.

These results are encouraging as they demonstrate that, using larger tile sizes (e.g. > 512), it is feasible to process a full S2 chip well within a 600 s window on COTS accelerator devices. We do not expect a significant improvement in speed reducing the input to 3 channels (i.e. RGB), as this only affects the number of operations performed on the first convolutional layer. The main limitation of these edge devices is available RAM and as we have shown, benchmarking is required to establish the optimal input size for a particular model^45^.

## Conclusions

As more and more EO missions are launched, there is a commensurate increase in the amount of data that must be sent back to Earth. Given that bandwidth is a costly resource, it is also prudent to consider ways to reduce wasteful data transfer and to prioritise critical information. Current applications of onboard ML have demonstrated that low-power platforms already have the capability to effectively filter low quality data, and to perform analysis of that data such as classification and segmentation. We expect that automating tasks, e.g. science target selection, will also become a possibility in the near future. By utilising onboard processing, we envision a hybrid EO strategy: sensing platforms (“eyes”) act in concert with in-orbit compute nodes (“brains”), prioritising data capture and transfer to the ground.

In this paper we articulated a vision for how machine learning could enhance remote sensing observations and we take the first practical steps to develop and test that vision. Supported by ESA, and in collaboration with our partners at D-Orbit and Unibap, we designed and successfully tested a machine learning payload that was launched into orbit in June 2021.

To summarize, these are some of the lessons that we have learned during the overall process:

1. Deploying a ML payload to segment a $10 k\times 10$ k pixels multi-band Sentinel 2 chip is eminently feasible in the power constrained computing environment of a small satellite.
2. Careful choice of ML algorithm, network architecture and pre-processing parameters (e.g., tile size) are required to extract the best performance from satellite hardware. In this work we focused on demonstrating feasibility; further optimizations could be tackled to reduce compute time if needed.
3. The Intel Movidius Myriad X processor is capable of good performance, as are the other ML accelerator devices (NVIDIA Jetson, Google Coral), but memory limitations impact what network architectures can run well (or indeed at all), and which are suitable for modification after launch.
4. While ML-inference processing times are consistent, time and effort to post-process the outputs into polygons can vary considerably, depending on the complexity of the segmentation masks. Additionally, our work does not take into account the timing required by the pre-processing steps of an optical payload such as Sentinel-2 (band-to-band alignement, radiometric correction and fine coregistration). This must be accounted for in any system design.
5. Data from low-resolution RGB cameras will likely be of limited value—on its own or as a trigger for tasking other space assets. Our results suggest that *WorldFloods* models rely heavily on the infrared bands for water detection (as expected).
6. The main motivator of the *WorldFloods* ML payload is to decrease the lag time to deliver flood maps by creating smaller data products for download. The compression factor for the data is typically between 200 and 10,000, but this is not guaranteed and it can be much smaller for complex scenes, or small images.
7. If the telecommunication link is no longer a limiting factor then the advantage of the *WorldFloods* payload is diminished. However, the demonstration of the ML processing capability is still valid.

## Data Availability

The Sentinel-2 dataset used on this work is a subset of the *WorldFloods* dataset published in Mateo-Garcia et al.^12^. Instructions to download the data can be found at https://spaceml-org.github.io/ml4floods/content/worldfloods_dataset.html.

## Code availability

The code to train models in the *WorldFloods* dataset together with all the architectures used in this work are available athttps://github.com/spaceml-org/ml4floods.
